# Work of the Southwestern Conference on Tuberculosis

**Published:** 1912-05

**Authors:** 


WORK OF THE SOUTHWESTERN CONFERENCE ON TUBERCULOSIS, held at Waco, Texas, April 16th (ult.). Report of this important meeting reached us too late for more than a brief outline of the work. The following resolutions were adopted. There was a large attendance of delegates, several States being represented.

A resolution declaring that institutions for the care of consumptives are necessary in the prevention of tuberculosis, and calling upon the Legislatures of Southwestern States to provide same.

A resolution advocating publicity as to the lack of free hospitals for stranger consumptives in the Southwest; the inability of charity organizations to aid such; the difficulty of securing suitable employment; that consumptives coming to the Southwest should have funds sufficient to carry them for about one year.

A resolution declaring that tuberculosis can not be eliminated without improving living and working conditions and describing legislation necessary to secure such improvement.

A resolution declaring the care of tuberculous strangers in the Southwest to be an interstate problem, and calling upon the Federal government to convert abandoned forts and military reservations in the Southwest into Tuberculosis Sanatoria.

Mrs. O. B. Colquitt presided. In addition to the above, she has circularized county judges, advocating free dispensaries and nurses for the indigent sick; the establishment of hospitals with separate provision for consumptives.

